# Characterization of Brain Iron Deposition Pattern and Its Association With Genetic Risk Factor in Alzheimer’s Disease Using Susceptibility-Weighted Imaging

**DOI:** 10.3389/fnhum.2021.654381

**Published:** 2021-06-07

**Authors:** Peiting You, Xiang Li, Zhijiang Wang, Huali Wang, Bin Dong, Quanzheng Li

**Affiliations:** ^1^Beijing International Center for Mathematical Research, Peking University, Beijing, China; ^2^Massachusetts General Hospital and Harvard Medical School, Boston, MA, United States; ^3^Peking University Institute of Mental Health (Sixth Hospital), Beijing, China; ^4^National Clinical Research Center for Mental Disorders and Key Laboratory of Mental Health, Ministry of Health, Peking University, Beijing, China; ^5^Beijing Municipal Key Laboratory for Translational Research on Diagnosis and Treatment of Dementia, Beijing, China

**Keywords:** Alzheimer’s disease, brain iron deposition, SWI, feature selection, genetic risk factor

## Abstract

The presence of iron is an important factor for normal brain functions, whereas excessive deposition of iron may impair normal cognitive function in the brain and lead to Alzheimer’s disease (AD). MRI has been widely applied to characterize brain structural and functional changes caused by AD. However, the effectiveness of using susceptibility-weighted imaging (SWI) for the analysis of brain iron deposition is still unclear, especially within the context of early AD diagnosis. Thus, in this study, we aim to explore the relationship between brain iron deposition measured by SWI with the progression of AD using various feature selection and classification methods. The proposed model was evaluated on a 69-subject SWI imaging dataset consisting of 24 AD patients, 21 mild cognitive impairment patients, and 24 normal controls. The identified AD progression-related regions were then compared with the regions reported from previous genetic association studies, and we observed considerable overlap between these two. Further, we have identified a new potential AD-related gene (MEF2C) closely related to the interaction between iron deposition and AD progression in the brain.

## Introduction

Alzheimer’s disease (AD) is among the leading causes of death in the United States and has been on the rising trend in the past decade with the aging populations (Alzheimer’s Association, 2011). More than 47 million people worldwide are estimated to have AD and related dementias. This number is expected to reach 152 million by 2050, with one new case of dementia diagnosed every 3 s (Patterson, 2018). As no effective treatment has been found to delay the onset and progression of AD (Selkoe, 2012), early diagnosis of AD and understanding of the progression from mild cognitive impairment (MCI) to AD is essential for preventative and therapeutic strategies (Gauthier et al., 2006).

Progression of AD can lead to structural and functional changes in the brain, which various imaging techniques can capture. Differential brain structural diagnostic markers derived from T1-weighted magnetic resonance imaging (MRI) have been reported for AD (Cuingnet et al., 2011), MCI (Driscoll et al., 2009), and MCI–AD conversion (Davatzikos et al., 2011) based on brain atrophy measurement (Jack et al., 2004) and its spatial pattern (Davatzikos et al., 2008). Diffusion MRI can measure white matter connectivity and microstructural integrity. It may also be supportive for the diagnosis of AD, based on both change in white matter tracts (Douaud et al., 2011) and global/local fractional anisotropy (FA) (Medina et al., 2006; Zhang et al., 2007). Functional magnetic resonance imaging (fMRI) has also been explored to characterize cognitive and behavior changes caused by AD progression (Li et al., 2019). Previous studies observed that disruption of resting-state functional networks could differentiate MCI/AD with normal controls (Rombouts et al., 2005); so does the decreased activation in cognition-related brain regions measured by memory encoding task (Machulda et al., 2003). Other functional imaging techniques, such as electroencephalography (EEG) and magnetoencephalography (MEG), have been demonstrated to detect the brain signal spectrum shift (Fernández et al., 2006) and coherence (Jeong, 2004). Previous studies also reported the utility of these techniques in modeling brain network alterations in MCI patients after cognitive training (Xu et al., 2020). Besides, PET imaging has been established as a standard approach to investigate pathological features and imaging biomarkers for AD, including neuritic plaques of amyloid-β peptide fibrils (Nordberg, 2004), hyper-phosphorylated tau neurofibrillary tangles (Ossenkoppele et al., 2016), as well as their respective propagation patterns (Sepulcre et al., 2018; Guo et al., 2019). Recently, the fusion of multiple imaging modalities for the early diagnosis of MCI and AD has been well studied and demonstrated improved performance over single-modality biomarkers (Zhang et al., 2011).

Among all potential imaging biomarkers for AD, one crucial marker is the excessive iron deposition in the brain. In vitro and in vivo studies have observed that excessive iron deposition in the brain might promote neurotoxicity, which causes neuronal injury and has been recognized as a putative factor in AD pathogenesis (Stankiewicz et al., 2007). Previous literature on the iron content measured with MR susceptibility-weighted imaging (SWI) (Halefoglu and Yousem, 2018) has reported significant iron deposition in brain regions related to brain cognitive and memory functions in AD, including substantia nigra, globus pallidum, hippocampus, putamen, and caudate nucleus. It has also been found that iron can induce the production and accumulation of amyloid-β plaques and bind to tau protein to induce tau protein phosphorylation aggregation (Liu et al., 2018). A meta-analysis on 1,813 AD patients and 2,401 normal controls concluded that specific brain regions had statistically significantly higher iron concentrations that can be related to AD (Tao et al., 2014). Besides, genetic factors have been found to play an essential role in the development of neurodegenerative disease in the context of iron deposition. It has been reported that the circulation of iron in the brain involves a complex interaction between metabolic and genetic processes (Rouault, 2013). It has been widely reported that genetic mutations can cause excessive iron deposition at the systemic level, posing as risk factors for several diseases such as acute myocardial infarction (Roest et al., 1999; Tuomainen et al., 1999). Similar gene mutations have also been reported to be related to neurodegenerative diseases (Hagemeier et al., 2012), for example, through the oxidative stress process that ultimately leads to the formation of neurofibrillary tangles senile plaques in AD (Crichton et al., 2011).

However, the predictive value of iron deposition to AD progression, especially with the current advancement of machine learning methods, is still unclear and largely understudied. With the aging of the brain, the excessive iron deposition could be related to many factors besides AD, such as increased permeability of the blood–brain barrier, dilation of blood vessels, redistribution of iron, and iron homeostasis changes (Ward et al., 2014). In the initial stage of AD patients, increasing iron is always along with β-amyloid peptide gathering, which provides the theoretical basis of MRI-based diagnosis (Ward et al., 2014). In addition, while region-specific iron deposition in the normal aging population has been investigated and observed in substantia nigra, putamen, globus pallidum, and caudate nuclei, iron-related pathogenic mechanisms are needed to explain the cause of such selectivity (Zecca et al., 2004). The regional heterogeneity and age-related brain iron have been confirmed by MRI (Zecca et al., 2004; Ramos et al., 2014).

Susceptibility-weighted imaging plays a vital role in the estimation of iron deposition (Sheelakumari et al., 2016). Thus, it could be used to detect abnormal iron deposition related to the progress of AD. Most diagnostic works using SWI imaging technology are currently based on manual or semi-manual measurement of the region of interest (ROI) in MRI images, which relies on previous knowledge and is usually confined to the hippocampus and entorhinal cortex (Zhang et al., 2015). In the past decade, with the advancement of machine learning methodologies, various computer-assisted models have been developed for the early diagnosis of AD, including SVM-based classifier (Davatzikos et al., 2011), Random Forest (Lebedev et al., 2014), and most recently, deep learning methods (Ortiz et al., 2016). However, there is little work on using machine learning methods to analyze iron deposition in the brain and facilitate automatic AD/MCI detection by SWI.

Thus, in this work, we used feature selection techniques and classification algorithms to analyze SWI image data acquired from a group of 69 subjects. After image pre-processing, iron deposition characteristics were extracted from SWI images based on different brain atlases. We then investigated the prediction power of different feature selection methods/atlas/classifier combinations. The best set of brain regions selected by the feature selection procedure were analyzed and compared to neuroscientific findings. We also obtained human gene expression data from the public Allen Human Brain Atlas Microarray dataset to investigate the relationship between iron deposition, genetic risk factor, and AD progression. This integrated analysis framework could lead us to answer the questions of (1) whether iron deposition as characterized by SWI images can be used to differentiate the three groups of subjects (healthy control, MCI, and AD), (2) which brain regions are involved in the differentiation, and (3) whether those brain regions have common genetic factors.

The organization of the rest of this paper is as follows: in the Materials and Methods section, we will introduce the SWI imaging dataset, its pre-processing pipeline, as well as the feature selection and classification methods used in this work. The Result section will showcase and discuss the classification performance and discriminative brain regions identified by the feature selection method. The identified brain regions are then combined with gene expression data to analyze their underlying relationship and discover new AD-related genes.

## Materials and Methods

### Study Population and Image Acquisition

All participants were recruited to establish a registry at the Dementia Care and Research Center, Peking University Institute of Mental Health. The clinical diagnosis of AD was made according to the International Classification of Disease, 10th Revision (ICD-10) (World Health Organization, 2004) and the criteria for probable AD of the National Institute of Neurological and Communicative Disorders and the Stroke/Alzheimer’s Disease and Related Disorders Association (NINCDS-ADRDA) (McKhann et al., 1984). The clinical diagnosis of MCI was made according to Petersen’s MCI criteria with the MMSE score of no less than 24. All the healthy controls had no history of neurological or psychiatric disorders, and subjective cognitive complaints or objectively abnormal cognitive assessment. Other inclusion criteria were as follows: age ≥55 years, right-handed, and primary school education (≥6 years). Exclusion criteria were as follows: current or previous neuropsychiatric diseases, such as Parkinson’s disease, epilepsy, alcohol or substance abuse/dependence, and head injury with loss of consciousness that could affect cognition or psychiatric behavior. This study was approved by the ethics committee of Peking University Institute of Mental Health (Sixth Hospital), Beijing, China. All participants were fully informed regarding the study protocol and provided written informed consent.

Participants were scanned on a 3-Tesla MR system (Siemens Magnetom Trio, A Tim System, Germany) using a standard 8-channel head coil at Peking University Third Hospital. T1-weighted magnetization-prepared rapidly acquired gradient-echo (MPRAGE) sequence was used to acquire high-resolution 3D MR anatomical images using the following parameters: repetition time (TR)/echo time (TE) = 2,530 ms/3.44 ms; time inversion (TI) = 1,100 ms; slice number = 192; slice thickness = 1.0 mm; gap = 0 mm; matrix = 256 × 256; field of view (FOV) = 256 mm × 256 mm; flip angle = 7°. SWI images were acquired using the following parameters: repetition time (TR)/echo time (TE) = 27 ms/20 ms; flip angle = 15°; slice thickness = 1.5 mm; voxel resolution = 0.8984 mm × 0.8984 mm × 1.5 mm. The Institutional Review Board of Peking University Sixth Hospital approved this study.

Similar to our previous genetic data studies on Allen Mouse Brain Atlas (AMBA) dataset (Li et al., 2017a, b), in this work, we used the Human Brain Atlas Microarray^1^ data (Shen et al., 2012) to search the anatomical brain regions associated with a specific gene. The microarray data provided by Human Brain Atlas Microarray includes the expression value (normalized Z-score) on each brain region from each subject (donor), where we would average these Z-scores across different subjects. In case there were multiple probes used to detect the same gene, Z-scores across different probes would also be averaged. After calculating the averaged Z-scores of the target gene, we applied a threshold of 0.3 to determine whether that gene is considered highly expressed in each brain region. Finally, we would obtain a list of “highly expressed” brain regions associated with it for each target gene, which could be further matched to the regions in SWI data.

### Image Preprocessing

Susceptibility-weighted imaging data were normalized into the MNI space based on transformation parameters derived from aligning T1 images to the MNI standard template using diffeomorphic anatomical registration through the exponentiated lie algebra (DARTEL) method (Ashburner, 2007) using Statistical Parametric Mapping (SPM12:^2^), then resampled to 1.5-mm isotropic voxels followed by spatially smoothing with a 6-mm full width at half maximum Gaussian kernel. We then applied three types of brain atlas: AAL (anatomical automatic labeling, 116 regions with 90 cerebral cortex and 26 cerebellar cortex regions), Harvard–Oxford (48 regions), and MMP (matrix metalloproteinase, 180 regions) to extract iron deposition information in the corresponding brain regions from SWI images. Specifically, each brain region (defined by one type of atlas) in the registered SWI images were characterized by the collection of voxels:

$$v_{i,j},i=1,\ldots ,T,j\in S$$

where *T* is the number of subjects in the dataset (69 in this study), *S* consists of the *R* number of regions in the atlas, *S* = 1,…,*S*_1_,*S*_1_ + 1,…,*S*_2_,…,*S*_*R*_. *S*_*k*_ denotes the number of voxels in the *k*-th region. In this way, we can obtain the phase value (i.e., iron content) of the *i*-th subject in the *k*-th region by

$$b_{i\,k}^{m\,e\,a\,n}=\frac{1}{S_{k}-S_{k-1}}\,\sum_{j=S_{k-1}+1}^{S_{k}}v_{i,j}$$

The final iron content vector for the *i*-th subject in the *k*-th region is then

$$X_{i\,k}=\frac{-b\times \pi }{4096},$$

where value in *X* varies from −*π* to *π*, and *b* varies from −4,096 to 4,095.

### Feature Selection and Classification

To identify the most discriminative brain regions toward classification of AD, MCI, and NC, we explored the commonly used supervised feature selection methods of Lasso and Adaptive Lasso for the analysis.

(1) Lasso: for dataset *D* = (*x*_1_,*y*_1_),(*x*_2_,*y*_2_),…,(*x*_*m*_,*y*_*m*_), where *x* ∈ *R^d^*,*y* ∈ *R*, in this work *x* denotes the iron content vector and *y* denotes the patient label (AD/MCI/NC), we consider the simple linear regression model with the squared error as a loss function:

$${\text{min}}_{w}\,\sum_{i=1}^{m}{\left(y_{i}-w^{T}\,x_{i}\right)}^{2}$$

When there are much more features than samples, the above equation is prone to be overfitting. To solve the problem, the regularization term is introduced. With *l*−1 norm regularization, the Lasso (Least Absolute Shrinkage and Selection Operator) algorithm is

$${\text{min}}_{w}\,\sum_{i=1}^{m}{\left(y_{i}-w^{T}\,x_{i}\right)}^{2}+\lambda \,{||w||}_{1},$$

with the regularization parameter λ>0.

(2) Adaptive Lasso: by adding weights to the penalty term in the original Lasso, the Adaptive Lasso can counteract the possible biased estimate in LASSO, with the following loss function:

$${\text{min}}_{w}\,\sum_{i=1}^{m}{\left(y_{i}-w^{T}\,x_{i}\right)}^{2}+\lambda \,\sum_{j}\frac{\left|\beta_{j}\right|}{w_{j}},$$

where *w_j_* = β*_j_(OLS)*.

Both Lasso and Adaptive Lasso can identify a subset of all regions (termed “regional feature”) in a given atlas by the non-zero weights found in regression. Iron content information from the selected regional features were then used to train various classifiers, including AdaBoost, LinearSVC, Randomtree, and XGBoost, to perform a three-class (healthy control/MCI/AD) classification. We set the basic classifier of AdaBoost algorithm to cycle 100 times with a learning rate of 0.1. The depth of Randomtree is four. For the XGBoost algorithm, we used the tree model with maximum depth of five and softmax as activation function.

## Results

**FIGURE 1:**
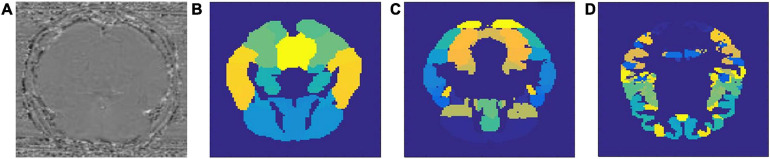
Sample SWI image **(A)** and three types of brain atlases used: **(B)** AAL atlas, **(C)** Harvard–Oxford atlas, **(D)** MMP atlas.

Results of this study are organized into two parts: in the first part we will describe the performance of the models we used for classifying AD and MCI patients from healthy controls. More importantly, we will analyze the region-specific feature extracted for making the classification. In the second part, we will connect the identified regions with gene expression data, both for the purpose of investigating the validity of the identified regions and to discover potentially new AD-related gene(s). A sample set of SWI images and the three different brain atlases used in this study are visualized in Figure 1.

### Classification Performance and the Regional Features Extracted

By using Lasso and adaptive Lasso to extract discriminative features (i.e., brain regions) on SWI images defined on three different types of brain atlas, we now obtained the AD-predictive regions for each feature selection method (Lasso/adaptive Lasso) and each atlas (AAL, Harvard–Oxford, MMP) combination. To investigate the discriminative power of the obtained AD-predictive regions, we designed a 10-fold cross-validation scheme where the dataset would be randomly divided into training/validation set (62 subjects) and testing set (7 subjects), where regional features based on feature selection method/atlas combination will be extracted correspondingly. Different classification methods would be then trained and tested on the extracted features in this cross-validation experiment. The experiment was repeated for 100 times, the average classification performance with regarding to different feature selection method (Lasso/Adaptive Lasso), atlas (AAD/Harvard/MMP), and classifiers (AdaBoost/LinearSVC/Randomtree/XGBoost) are summarized in Table 1.

**TABLE 1:** Classification accuracy of different classifiers (listed in each row) on different atlases (listed in each column), based on the regional feature selected by Lasso or Adaptive Lasso Highest classification accuracies achieved for Lasso and Adaptive Lasso are marked by bold.

	**Lasso**			**Adaptive Lasso**		
**Classifier**	**AAL**	**Harvard**	**MMP**	**AAL**	**Harvard**	**MMP**
Adaboost	0.3145	0.3116	0.4304	0.3665	0.4415	0.4126
LinearSVC	**0.7388**	0.3578	0.3939	**0.7157**	0.5382	0.5497
Randomtree	0.4141	0.3564	0.3448	0.4747	0.4098	0.4415
XGBoost	0.4242	0.4227	0.404	0.4862	0.4747	0.518

**FIGURE 2:**
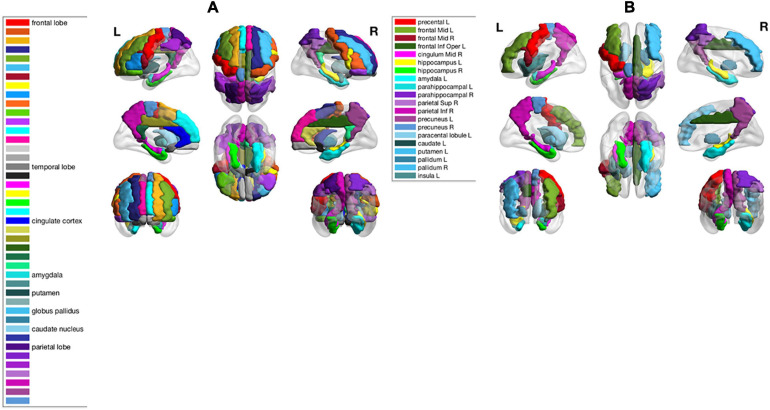
**(A)** Names of 8 brain regions reported in Tao et al. (2014) and their visualizations as colored brain surface areas, according to the color bar to the left. **(B)** Names of the selected brain regions by Lasso algorithm in this study. The brain regions were visualized using the BrainNet Viewer (Xia et al., 2013).

As shown in the performance matrices, the best classification accuracy (0.7388) was obtained by the LinearSVC classifier on AAL atlas, using Lasso for the feature selection. Thus, in later analysis, we will investigate the feature regions and the corresponding neuroscience implications based on Lasso feature selection on AAL atlas. In total, 20 AAL regions that were selected as discriminative features by Lasso are listed and visualized as colored brain surfaces in Figure 2B. In addition, a meta-analysis study in Tao et al. (2014) have found that eight brain regions are closely related to AD, including frontal lobe (FL), parietal lobe (PL), temporal lobe, amygdala (Amg), putamen, cingulate cortex, globus pallidus (GP), and caudate nucleus, which are listed and visualized in Figure 2A.

### Analysis of Gene Expression Distribution on Selected Regional Features

The AD-predictive regions identified by our models were then compared with the brain regions associated with three commonly known AD risk factor genes (APOE, MAPT, and CLU). As reported in our previous study, apolipoprotein E (APOE) genotype has been found to account for the majority of AD risk and pathology (Marioni et al., 2017; Sepulcre et al., 2018). Microtubule-associated protein tau (MAPT) gene is related to the encoding of tau protein and can cause potential vulnerability to tau accumulation as found in our work (Sepulcre et al., 2018), leading to frontotemporal dementia-spectrum (FTD-s) disorders (Coppola et al., 2012). The clusterin gene (CLU) has been reported to be associated with degraded regional cerebral blood flow (Thambisetty et al., 2013) and white matter integrity (Braskie et al., 2011). Regions that are highly expressed by APOE, MAPT, and CLU were identified from Allen Human Brain Atlas Microarray data based on the thresholding of their normalized Z-Scores as previously introduced. The names of these regions, along with the AD-predictive regions identified in this work, are summarized in Table 2.

**TABLE 2:** List of regions identified by our method as predicative to AD (first column), as well as regions associated with APOE, MAPT, and CLU gene.

**Regions identified by our method**	**Regions asscociated with APOE**	**Regions asscociated with MAPT**	**Regions asscociated with CLU**
amygdala!	amygdala	cingulate gyrus	amygdala
caudate nucleus.L	dorsal thalamus	frontal lobe	cingulate gyrus
hippocampus!	globus pallidus	insula	dorsal thalamus
hippocampus.R	hypothalamus	occipital lobe	frontal lobe
inferior frontal gyrus, opercular part.L	striatum	parahippocampal gyms	globus pallidus
inferior parietal, supramarginal and angular gyri.R	subthalamus	parietal lobe	insula
insula!	ventral thalamus	temporal lobe	occipital lobe
lenticular nucleus, putamen.L			parietal lobe
median cingulate and paracingulate gyri.R			striatum
middle frontal gyrus.L			temporal lobe
middle frontal gyrus.R			
pallidum!			
pallidum.R			
paracental Lobule!			
parahippocampal!			
parahippocampal.R			
precentral gyms!			
precuneus!			
precuneus.R			
superior parietal gyrus.R			

In addition to these three genes, we preselected a total of 21 genes based on the literature reports on aging, dementia, and MCI/AD progression, and obtained their correspondingly highly expressed regions in the Allen Human Brain Atlas. Based on the premise that the 20 AD-predictive regions as identified in this work from SWI data are associated with AD both at imaging and genetic level, we investigated how frequent each of the 21 genes are expressed in these regions, and used the derived gene presence frequency as a measurement for the association between each gene and AD development. However, the brain region definition used in the Allen Human Brain Atlas (shown as “top-level structure name” in the downloaded expression data) is different from the AAL atlas used in this work, where the top-level structures are usually larger and can cover multiple regions in the AAL atlas. Thus, we firstly identified a total of 18 top-level structures from the microarray data with higher expressions for either of these 21 genes (i.e., the union of highly expressed regions), including structures of FL, cingulate gyrus (CgG), hippocampus formation (HiF), parahippocampal gyrus (PHG), PL, Amg, GP, and striatum (Str). As shown in Table 3, each gene (row) has its corresponding normalized Z-score at each top-level structure (column). After that, we mapped these top-level structures with the regions in AAL atlas by comparing their spatial distributions in the MNI space. Based on this many-to-many mapping, we can find which AAL region(s) are included in each top-level structure. Between the 18 top-level structures and the 20 AD-predictive regions, we obtained the following 18 × 1 weight vector [4,1,1,2,2,0,2,0,5,0,1,0,2,2,0,0,0,0,0,0]. Each value in the vector indicates how many of the 20 AD-predictive region(s) are presented in that top-level structure, for example, the first value of four indicates that the top-level structure of FL includes four AD-predictive regions. Finally, we can calculate the presence frequency for each gene by multiplying the normalized Z-score in each top-level structure with the corresponding weight value and adding them together, as listed in the “Frequency” column in Table 3. Higher value of presence frequency indicates that the target gene in overall is more frequently expressed in regions that are predictive to AD, thus could be potentially more associated with AD.

**TABLE 3:** Calculated gene presence frequency (in column “Frequency”) from the 21 preselected genes as listed in each row.

**Gene**	**Frequency**	**FL**	**Ins**	**CgG**	**HP**	**PHG**	**OL**	**PL**	**TL**	**Amy**	**BF**	**GP**	**Str**	**Cla**	**ET**	**HT**	**ST**	**DT**	**VT**
MART	**12.52**	1.5	1.5	1.3	0.29	0.14	1.2	1.4	1.5	–0.48	0.18	–0.67	–1.3	0.11	–2.2	–0.36	–0.45	–0.09	–0.85
APOE	–2.86	–0.87	–0.63	–0.57	–1.2	–0.03	–0.48	–0.79	–0.58	1	1.4	2.4	1.2	–0.48	0.34	0.71	0.38	1	0.8
PICALM	–11.88	−1	–1.3	–0.94	–0.39	–0.76	–0.75	–0.9	−1	0.04	0.74	0.97	–0.33	0.4	–0.75	–0.61	0	–0.06	2
BIN1	–5.42	–0.55	–0.6	–0.39	0.53	–0.67	–0.42	–0.51	–0.47	–0.06	0.65	0.88	–0.55	0.2	–2.1	–1.3	0.45	0.32	1.4
CLU	**14.91**	0.61	0.76	0.73	0.76	0.48	0.51	0.55	0.73	0.55	0.86	0.25	2.4	0.13	–1.2	–0.13	–0.29	0.02	–0.57
CR1	–5.62	–0.24	–0.25	–0.12	–1.1	0.19	–0.68	–0.04	0.39	–0.25	–1.1	–0.62	–0.38	–0.43	–0.87	–0.96	0.4	–0.55	1.4
ABCA7	–15.14	–0.7	−1	–0.55	1	–1.6	–0.24	–0.8	–0.94	0.93	1.6	–1.5	–1.8	0.84	–0.49	–0.48	1.4	0.11	0.73
SORL1	–1.75	0.31	–0.38	–0.07	0.31	–0.6	0.93	0.56	–0.08	–1.6	–1.1	0.79	–2.4	–0.12	0.73	–0.95	0.8	–0.31	0.85
PLEKHC1	–5.22	–0.97	–0.84	–0.47	–0.85	0.04	–0.94	–0.83	–0.79	1.6	1.1	1.8	0.26	0.79	–0.19	0.15	0.48	0.34	1.2
CD2AP	–10.16	−1	–1.5	–1.3	–0.59	–0.67	–0.01	–0.59	–0.83	0.08	–1.3	0.62	0.4	–1.1	1.1	0.17	–0.12	0.52	–0.1
CD33	–11.74	–0.86	–0.85	–1.2	–0.76	–0.31	–1.4	–0.95	–0.65	0.54	–0.41	0.86	–0.79	0.57	0.36	–0.49	1.2	–0.32	1.3
APP	0.12	–0.29	–0.43	–0.12	0.42	0.16	0.09	–0.05	–0.2	0.7	0.01	0.47	–0.36	2	–1.1	–1.1	–0.29	0.11	1.3
PSEN1	–10.81	–0.56	–0.82	–0.56	–1.1	–0.98	–0.75	–0.68	–0.66	−1	0.66	1.1	–0.34	–0.59	–0.61	–1.1	0.41	–0	1.3
PSEN2	–14.85	–0.86	–0.79	–0.77	–0.43	–0.75	–1.3	–0.98	–0.85	–0.23	0.8	–0.5	–0.68	1.8	–0.93	0.59	1.5	0.83	0.77
CASS	–3.31	–0.36	–0.28	–1.1	–0.88	–0.37	–0.64	–0.75	0.41	0.14	1.2	1.8	0.97	–0.77	–1.7	0.89	–0.51	0.83	0.77
EPHA1	–3.68	–0.21	–0.31	–0.29	–0.48	0.05	–0.53	0.29	0.03	–0.05	–0.6	–0.9	–0.49	0.4	–0.72	–0.86	0.07	–0.32	0.78
PTK2B	7.19	0.58	0.35	0.36	2.2	–0.21	0.4	0.54	0.34	1.6	0.54	–1.6	–0.51	0.73	–1.8	–1.5	–0.75	0.89	0.73
INPP5D	–10.02	–0.89	–0.82	–1.1	–1.2	–1.1	0.43	–0.68	–0.77	–0.43	0.21	1.5	0.43	–0.49	1.7	0.04	1.1	–0.12	0.47
MEF2C	**14.59**	1.3	1.4	1.3	–0.21	1.1	1.4	1.4	1.4	0.64	–0.35	–0.73	–0.66	1.5	–0.81	–0.8	–0.73	–0.74	–0.4
CUGBP1	11.38	0.34	0.18	0.48	1.7	0.76	0.93	0.61	0.42	1.6	0.84	–0.64	0.55	1.8	–0.52	–0.48	–1.1	0.82	–0.46
MAAD	4.38	–0.37	–0.41	0.27	1.6			0.04	–0.15	2.3	0.48	–0.98	0.3	2.1	–0.96	–0.59	–0.01	–0.72	1.3

*Normalized Z-scores of each gene are provided in the corresponding top-level structure (column). A higher value indicates gene located in that structure has higher expression. Three genes with the highest presence frequencies (MAPT, CLU, and MEF2C) are highlighted in bold.*

## Discussion and Conclusion

In this study of analyzing the association between the development AD and iron deposition as characterized by SWI images, we used Lasso family algorithms for supervised dimensionality reduction to identify important regions that are discriminative to AD to overcome the challenge of small sample size and large feature number. We then applied different classification methods to investigate the diagnostic capability of SWI towards MCI and AD. Ten-fold cross-validation experiment results show that >70% accuracy can be achieved for this three-class classification task. Further investigation into the identified AD-related regions revealed that they are consistent with previous literature reports. The regions identified in this work cover all the eight regions previously reported.

We then co-analyzed the SWI-derived imaging features with the genetic data provided by Allen’s brain atlas. We found that the regions identified by the feature selection method are identical with the regions rich in gene expression associated with protein precipitation and the blood–brain barrier, as measured by the microarray data (Shen et al., 2012). Specifically, our study has found that:

(1) AD-predicative regions identified in this work cover most of the APOE-associated regions except for the dorsal thalamus and striatum. Iron is involved in the formation of astrocytes that might affect the permeability of the blood–brain barrier. It has been reported that AD patients have a breakdown of the blood–brain barrier before dementia, neurodegenerative diseases, and brain atrophy. APOE gene has been found to be the strongest AD risk gene involved in the damage of the blood–brain barrier (Montagne et al., 2015). On the other hand, the UCLA team (Raven et al., 2013) used FDRI to detect cerebral iron and studied the difference of iron content in the hippocampus and thalamus regions. As detected in our study, the iron levels increase at the hippocampus, not the thalamus, might be linked to an injury to the hippocampus.
(2) Our identified regions also include CLU/MAPT-associated regions except for the striatum, as well as the occipital lobe, which is commonly known as non-specific to AD. In the initial lesion regions of AD patients, increased iron concentration was associated with the accumulation of Aβ (amyloid β) and *tau* protein. Our previous study in Sepulcre et al. (2018) shows that CLU and MAPT genes are responsible for the high expression of Aβ and *tau* protein, respectively. Studies have shown that iron deposition was detected in microglia and astrocytes in the amygdala, and ferritin concentrations increase with age (Zecca et al., 2004). A study of 143 healthy individuals shows that iron deposition in the caudate nucleus increases with age, peaking at age 60 (Wang et al., 2012). Other literature reported similar iron increases with age in the putamen, globus pallidus, and caudate nucleus (Ward et al., 2014). Further studies on iron deposition in AD, MCI, and NC also revealed significant differences in the caudate nucleus and putamen (Wang et al., 2013).
(3) From the gene expression frequency analysis, our study observed that the top three genes presented in the identified AD-predictive regions are CLU, MEF2C, and MAPT. Besides the two previously reported AD-related genes (CLU/MAPT), the MEF2C gene plays a key role in the development of multiple types of tissues. It is currently known to be related to epilepsy, autism, and mental retardation (Rashid et al., 2014). However, its role in the adult brain is largely understudied. Recent evidence suggests that the MEF2C gene regulates memory forming structures (Cole et al., 2012), which implies its potential role in the memory degradation of AD patients.

There are several limitations of the current study, both on the method design and the data used. Specifically, the current feature selection and classification scheme are relatively simple due to the limited sample size. With a larger dataset, we can try more advanced data analytics methods such as deep learning to better map imaging features and disease development. We also recognized that the regional features identified in the current study are limited because the AAL atlas is relatively coarse for the detailed spatial analysis. In later studies, we will try more fine-grained parcellation of the brain or performing voxel-level analysis.

Our conclusions on the effectiveness of using SWI for AD diagnosis need to be validated by external datasets. Nevertheless, in this study, we have only collected susceptibility-weighted images as one single dataset. We have implemented the complete feature selection and classification pipeline into an integrated framework. We will publish the code onto a public repository so that external researchers can use the same regional features to test their prediction power and compare the classification performance. The possible role of the MEF2C gene also needs to be validated, both by testing the consistency of its expression in the MCI/AD population on another dataset other than the Allen brain atlas and by exploring the biological pathway of the MEF2C’s expression using bioinformatics tools.

## Data Availability Statement

The raw data supporting the conclusions of this article will be made available by the authors, without undue reservation.

## Ethics Statement

The studies involving human participants were reviewed and approved by Institutional Review Board of Peking University Sixth Hospital. The patients/participants provided their written informed consent to participate in this study.

## Author Contributions

PY was responsible for the model implementation, experiment, and manuscript writing. XL was responsible for the experiment design, image processing, and manuscript writing. ZW was responsible for the data curation and image processing of this work. HW, BD, and QL were responsible for the funding acquisition, project administration, and supervision of this work. All authors contributed to the article and approved the submitted version.

## Conflict of Interest

The authors declare that the research was conducted in the absence of any commercial or financial relationships that could be construed as a potential conflict of interest.

## Footnotes

1 https://human.brain-map.org/microarray/search

2 http://www.fil.ion.ucl.ac.uk/spm

## References

[B1] Alzheimer’s Association. (2011). 2011 Alzheimer’s disease facts and figures. *Alzheimers Dement.* 7 208–244. 10.1016/j.jalz.2011.02.004 21414557

[B2] Ashburner J. (2007). A fast diffeomorphic image registration algorithm. *NeuroImage* 38 95–113. 10.1016/j.neuroimage.2007.07.007 17761438

[B3] Braskie M. N. Jahanshad N. Stein J. L. Barysheva M. McMahon K. L. de Zubicaray G. I. (2011). Common Alzheimer’s disease risk variant within the CLU gene affects white matter microstructure in young adults. *J. Neurosc.* 31:6764. 10.1523/jneurosci.5794-10.2011 21543606 PMC3176803

[B4] Cole C. J. Mercaldo V. Restivo L. Yiu A. P. Sekeres M. J. Han J. H. (2012). MEF2 negatively regulates learning-induced structural plasticity and memory formation. *Nat. Neurosci.* 15 1255–1264. 10.1038/nn.3189 22885849

[B5] Coppola G. Chinnathambi S. Lee J. J. Dombroski B. A. Baker M. C. Soto-Ortolaza A. I. (2012). Evidence for a role of the rare p.A152T variant in MAPT in increasing the risk for FTD-spectrum and Alzheimer’s diseases. *Hum. Mol. Genet.* 21 3500–3512. 22556362 10.1093/hmg/dds161 PMC3392107

[B6] Crichton R. R. Dexter D. T. Ward R. J. (2011). Brain iron metabolism and its perturbation in neurological diseases. *J. Neural Transm.* 118 301–314. 10.1007/s00702-010-0470-z 20809066

[B7] Cuingnet R. Gerardin E. Tessieras J. Auzias G. Lehéricy S. Habert M.-O. (2011). Automatic classification of patients with Alzheimer’s disease from structural MRI: a comparison of ten methods using the ADNI database. *NeuroImage* 56 766–781. 10.1016/j.neuroimage.2010.06.013 20542124

[B8] Davatzikos C. Bhatt P. Shaw L. M. Batmanghelich K. N. Trojanowski J. Q. (2011). Prediction of MCI to AD conversion, via MRI, CSF biomarkers, and pattern classification. *Neurobiol. Aging* 32 2322.e19–27. 10.1016/j.neurobiolaging.2010.05.023 20594615 PMC2951483

[B9] Davatzikos C. Fan Y. Wu X. Shen D. Resnick S. M. (2008). Detection of prodromal Alzheimer’s disease via pattern classification of magnetic resonance imaging. *Neurobiol. Aging* 29 514–523. 10.1016/j.neurobiolaging.2006.11.010 17174012 PMC2323584

[B10] Douaud G. Jbabdi S. Behrens T. E. J. Menke R. A. Gass A. Monsch A. U. (2011). DTI measures in crossing-fibre areas: increased diffusion anisotropy reveals early white matter alteration in MCI and mild Alzheimer’s disease. *NeuroImage* 55 880–890. 10.1016/j.neuroimage.2010.12.008 21182970 PMC7116583

[B11] Driscoll I. Davatzikos C. An Y. Wu X. Shen D. Kraut M. (2009). Longitudinal pattern of regional brain volume change differentiates normal aging from MCI. *Neurology* 72 1906–1913. 10.1212/wnl.0b013e3181a82634 19487648 PMC2690968

[B12] Fernández A. Hornero R. Mayo A. Poza J. Gil-Gregorio P. Ortiz T. (2006). MEG spectral profile in Alzheimer’s disease and mild cognitive impairment. *Clin. Neurophysiol.* 117 306–314. 10.1016/j.clinph.2005.10.017 16386951

[B13] Gauthier S. Reisberg B. Zaudig M. Petersen R. C. Ritchie K. Broich K. (2006). Mild cognitive impairment. *Lancet* 367 1262–1270. 16631882 10.1016/S0140-6736(06)68542-5

[B14] Guo J. Qiu W. Li X. Zhao X. Guo N. Li Q. (2019). “Predicting Alzheimer’s disease by hierarchical graph convolution from positron emission tomography imaging,” in *Proceedings of the 2019 IEEE International Conference on Big Data (Big Data)* (Los Angeles, CA), 5359–5363.

[B15] Hagemeier J. Geurts J. J. G. Zivadinov R. (2012). Brain iron accumulation in aging and neurodegenerative disorders. *Expert Rev. Neurother.* 12 1467–1480. 10.1586/ern.12.128 23237353

[B16] Halefoglu A. M. Yousem D. M. (2018). Susceptibility weighted imaging: clinical applications and future directions. *World J. Radiol.* 10 30–45. 10.4329/wjr.v10.i4.30 29849962 PMC5971274

[B17] Jack C. R. Shiung M. M. Gunter J. L. O’Brien P. C. Weigand S. D. Knopman D. S. (2004). Comparison of different MRI brain atrophy rate measures with clinical disease progression in AD. *Neurology* 62 591–600. 10.1212/01.wnl.0000110315.26026.ef 14981176 PMC2730165

[B18] Jeong J. (2004). EEG dynamics in patients with Alzheimer’s disease. *Clin. Neurophysiol.* 115 1490–1505. 15203050 10.1016/j.clinph.2004.01.001

[B19] Lebedev A. V. Westman E. Van Westen G. J. P. Kramberger M. G. Lundervold A. Aarsland D. (2014). Random Forest ensembles for detection and prediction of Alzheimer’s disease with a good between-cohort robustness. *NeuroImage* 6 115–125. 10.1016/j.nicl.2014.08.023 25379423 PMC4215532

[B20] Li X. Guo N. Li Q. (2019). Functional neuroimaging in the new era of big data. *Genomics Proteom. Bioinform.* 17 393–401. 10.1016/j.gpb.2018.11.005 31809864 PMC6943787

[B21] Li Y. Chen H. Jiang X. Li X. Lv J. Li M. (2017a). Transcriptome architecture of adult mouse brain revealed by sparse coding of genome-wide in situ hybridization images. *Neuroinformatics* 15 285–295. 10.1007/s12021-017-9333-1 28608010 PMC5540769

[B22] Li Y. Chen H. Jiang X. Li X. Lv J. Peng H. (2017b). Discover mouse gene coexpression landscapes using dictionary learning and sparse coding. *Brain Struct. Funct.* 222 4253–4270. 10.1007/s00429-017-1460-9 28664394

[B23] Liu J.-L. Fan Y.-G. Yang Z.-S. Wang Z.-Y. Guo C. (2018). Iron and Alzheimer’s disease: from pathogenesis to therapeutic implications. *Front. Neurosci.* 12:632. 10.3389/fnins.2018.00632 30250423 PMC6139360

[B24] Machulda M. M. Ward H. A. Borowski B. Gunter J. L. Cha R. H. O’Brien P. C. (2003). Comparison of memory fMRI response among normal, MCI, and Alzheimer’s patients. *Neurology* 61 500–506. 10.1212/01.wnl.0000079052.01016.78 12939424 PMC2744465

[B25] Marioni R. E. Campbell A. Hagenaars S. P. Nagy R. Amador C. Hayward C. (2017). Genetic stratification to identify risk groups for Alzheimer’s disease. *J. Alzheimers Dis.* 57 275–283. 10.3233/jad-161070 28222519 PMC5345653

[B26] McKhann G. Drachman D. Folstein M. Katzman R. Price D. Stadlan E. M. (1984). Clinical diagnosis of Alzheimer’s disease: report of the NINCDS-ADRDA Work Group under the auspices of department of health and human services task force on Alzheimer’s disease. *Neurology* 34 939–944. 10.1212/wnl.34.7.939 6610841

[B27] Medina D. deToledo-Morrell L. Urresta F. Gabrieli J. D. E. Moseley M. Fleischman D. (2006). White matter changes in mild cognitive impairment and AD: a diffusion tensor imaging study. *Neurobiol. Aging* 27 663–672. 10.1016/j.neurobiolaging.2005.03.026 16005548

[B28] Montagne A. Barnes S. R. Sweeney M. D. Halliday M. R. Sagare A. P. Zhao Z. (2015). Blood-brain barrier breakdown in the aging human hippocampus. *Neuron* 85 296–302. 10.1016/j.neuron.2014.12.032 25611508 PMC4350773

[B29] Nordberg A. (2004). PET imaging of amyloid in Alzheimer’s disease. *Lancet Neurol.* 3 519–527. 15324720 10.1016/S1474-4422(04)00853-1

[B30] Ortiz A. Munilla J. Górriz J. M. Ramírez J. (2016). Ensembles of deep learning architectures for the early diagnosis of the Alzheimer’s disease. *Int. J. Neural Syst.* 26 1650025. 10.1142/s0129065716500258 27478060

[B31] Ossenkoppele R. Schonhaut D. R. Schöll M. Lockhart S. N. Ayakta N. Baker S. L. (2016). Tau PET patterns mirror clinical and neuroanatomical variability in Alzheimer’s disease. *Brain* 139 1551–1567. 10.1093/brain/aww027 26962052 PMC5006248

[B32] Patterson C. (2018). *World Alzheimer Report 2018: The State of the art of Dementia Research: New Frontiers.* London: Alzheimer’s Disease International (ADI), 32–36.

[B33] Ramos P. Santos A. Pinto N. R. Mendes R. Magalhães T. Almeida A. (2014). Iron levels in the human brain: a post-mortem study of anatomical region differences and age-related changes. *J. Trace Elem. Med. Biol.* 28 13–17. 10.1016/j.jtemb.2013.08.001 24075790

[B34] Rashid A. J. Cole C. J. Josselyn S. A. (2014). Emerging roles for MEF2 transcription factors in memory. *Genes Brain Behav.* 13 118–125. 10.1111/gbb.12058 23790063

[B35] Raven E. P. Lu P. H. Tishler T. A. Heydari P. Bartzokis G. (2013). Increased iron levels and decreased tissue integrity in hippocampus of Alzheimer’s disease detected in vivo with magnetic resonance imaging. *J. Alzheimers Dis.* 37 127–136. 10.3233/jad-130209 23792695

[B36] Roest M. van der Schouw Y. T. de Valk B. Marx J. J. Tempelman M. J. de Groot P. G. (1999). Heterozygosity for a hereditary hemochromatosis gene is associated with cardiovascular death in women. *Circulation* 100 1268–1273. 10491369 10.1161/01.cir.100.12.1268

[B37] Rombouts S. A. R. B. Barkhof F. Goekoop R. Stam C. J. Scheltens P. (2005). Altered resting state networks in mild cognitive impairment and mild Alzheimer’s disease: an fMRI study. *Hum. Brain Mapp.* 26 231–239. 10.1002/hbm.20160 15954139 PMC6871685

[B38] Rouault T. A. (2013). Iron metabolism in the CNS: implications for neurodegenerative diseases. *Nat. Rev. Neurosci.* 14 551–564. 10.1038/nrn3453 23820773

[B39] Selkoe D. J. (2012). Preventing Alzheimer’s disease. *Science* 337 1488–1492. 22997326 10.1126/science.1228541

[B40] Sepulcre J. Grothe M. J. d’Oleire Uquillas F. Ortiz-Terán L. Diez I. Yang H.-S. (2018). Neurogenetic contributions to amyloid beta and tau spreading in the human cortex. *Nat. Med.* 24 1910–1918. 10.1038/s41591-018-0206-4 30374196 PMC6518398

[B41] Sheelakumari R. Madhusoodanan M. Radhakrishnan A. Ranjith G. Thomas B. (2016). A potential biomarker in amyotrophic lateral sclerosis: can assessment of brain iron deposition with swi and corticospinal tract degeneration with DTI help? *AJNR. American journal of neuroradiology.* 37 252–258. 10.3174/ajnr.a4524 26494694 PMC7959949

[B42] Shen E. H. Overly C. C. Jones A. R. (2012). The allen human brain atlas: comprehensive gene expression mapping of the human brain. *Trends Neurosci.* 35 711–714. 23041053 10.1016/j.tins.2012.09.005

[B43] Stankiewicz J. Panter S. S. Neema M. Arora A. Batt C. E. Bakshi R. (2007). Iron in chronic brain disorders: imaging and neurotherapeutic implications. *Neurotherapeutics* 4 371–386. 10.1016/j.nurt.2007.05.006 17599703 PMC1963417

[B44] Tao Y. Wang Y. Rogers J. T. Wang F. (2014). Perturbed iron distribution in Alzheimer’s disease serum, cerebrospinal fluid, and selected brain regions: a systematic review and meta-analysis. *J. Alzheimers Dis.* 42 679–690. 10.3233/jad-140396 24916541

[B45] Thambisetty M. Beason-Held L. L. An Y. Kraut M. Nalls M. Hernandez D. G. (2013). Alzheimer risk variant clu and brain function during aging. *Biol. Psychiatry* 73 399–405. 10.1016/j.biopsych.2012.05.026 22795969 PMC3488132

[B46] Tuomainen T. P. Kontula K. Nyyssönen K. Lakka Timo A. Heliö T. Salonen Jukka T. (1999). Increased risk of acute myocardial infarction in carriers of the hemochromatosis gene Cys282Tyr mutation. *Circulation* 100 1274–1279. 10.1161/01.cir.100.12.1274 10491370

[B47] Wang D. Szyf M. Benkelfat C. Provençal N. Turecki G. Caramaschi D. (2012). Peripheral SLC6A4 DNA methylation is associated with in vivo measures of human brain serotonin synthesis and childhood physical aggression. *PLoS One* 7:e39501. 10.1371/journal.pone.0039501 22745770 PMC3379993

[B48] Wang D. Zhu D. Wei X. E. Li Y. H. Li W. B. (2013). Using susceptibility-weighted images to quantify iron deposition differences in amnestic mild cognitive impairment and Alzheimer’s disease. *Neurol. India* 61 26–34. 10.4103/0028-3886.107924 23466836

[B49] Ward R. J. Zucca F. A. Duyn J. H. Crichton R. R. Zecca L. (2014). The role of iron in brain ageing and neurodegenerative disorders. *Lancet Neurol.* 13 1045–1060. 10.1016/s1474-4422(14)70117-6 25231526 PMC5672917

[B50] World Health Organization. (2004). *International Statistical Classification of Diseases and Related Health Problems: Tabular List.* Geneva: World Health Organization.

[B51] Xia M. Wang J. He Y. (2013). BrainNet viewer: a network visualization tool for human brain connectomics. *PLoS One* 8:e68910. 10.1371/journal.pone.0068910 23861951 PMC3701683

[B52] Xu M. Wang Z. Zhang H. Pantazis D. Wang H. Li Q. (2020). A new Graph Gaussian embedding method for analyzing the effects of cognitive training. *PLoS Comput. Biology.* 16:e1008186. 10.1371/journal.pcbi.1008186 32941425 PMC7524000

[B53] Zecca L. Stroppolo A. Gatti A. Tampellini D. Toscani M. Gallorini M. (2004). The role of iron and copper molecules in the neuronal vulnerability of locus coeruleus and substantia nigra during aging. *Proc. Natl. Acad. Sci. U.S.A.* 101 9843–9848. 10.1073/pnas.0403495101 15210960 PMC470762

[B54] Zhang D. Wang Y. Zhou L. Yuan H. Shen D. (2011). Multimodal classification of Alzheimer’s disease and mild cognitive impairment. *NeuroImage* 55 856–867. 21236349 10.1016/j.neuroimage.2011.01.008 PMC3057360

[B55] Zhang Y. Dong Z. Phillips P. Wang S. Ji G. Yang J. (2015). Detection of subjects and brain regions related to Alzheimer’s disease using 3D MRI scans based on eigenbrain and machine learning. *Front. Comput. Neurosci.* 9:66. 10.3389/fncom.2015.00066 26082713 PMC4451357

[B56] Zhang Y. Schuff N. Jahng G.-H. Bayne W. Mori S. Schad L. (2007). Diffusion tensor imaging of cingulum fibers in mild cognitive impairment and Alzheimer disease. *Neurology* 68 13–19. 10.1212/01.wnl.0000250326.77323.01 17200485 PMC1941719

